# Sex Hormones Regulate Innate Immune Cells and Promote Sex Differences in Respiratory Virus Infection

**DOI:** 10.3389/fimmu.2018.01653

**Published:** 2018-07-20

**Authors:** Sapana Kadel, Susan Kovats

**Affiliations:** ^1^Arthritis & Clinical Immunology Program, Oklahoma Medical Research Foundation, Oklahoma City, OK, United States; ^2^Department of Microbiology and Immunology, University of Oklahoma Health Sciences Center, Oklahoma City, OK, United States

**Keywords:** sex hormones, respiratory virus, lung, estrogen, androgen, innate immunity

## Abstract

Sex differences in the incidence and severity of respiratory virus infection are widely documented in humans and murine models and correlate with sex biases in numbers and/or functional responses of innate immune cells in homeostasis and lung infection. Similarly, changes in sex hormone levels upon puberty, pregnancy, and menopause/aging are associated with qualitative and quantitative differences in innate immunity. Immune cells express receptors for estrogens (ERα and ERβ), androgens (AR), and progesterone (PR), and experimental manipulation of sex hormone levels or receptors has revealed that sex hormone receptor activity often underlies sex differences in immune cell numbers and/or functional responses in the respiratory tract. While elegant studies have defined mechanistic roles for sex hormones and receptors in innate immune cells, much remains to be learned about the cellular and molecular mechanisms of action of ER, PR, and AR in myeloid cells and innate lymphocytes to promote the initiation and resolution of antiviral immunity in the lung. Here, we review the literature on sex differences and sex hormone regulation in innate immune cells in the lung in homeostasis and upon respiratory virus infection.

## Introduction

**Figure 1:**
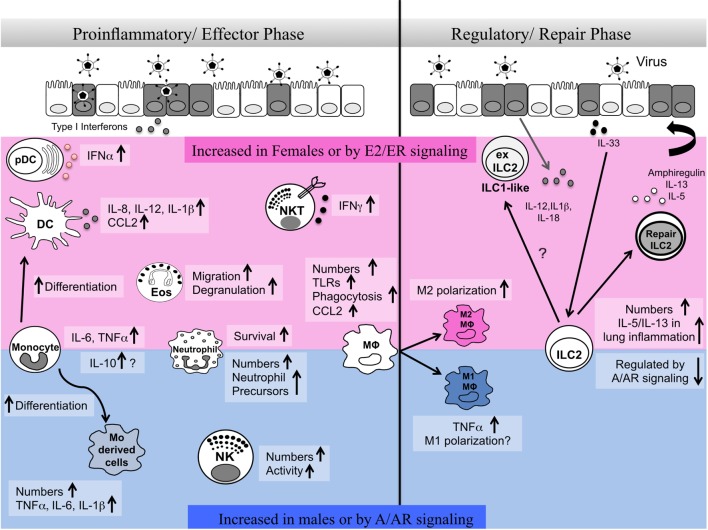
Sex differences in innate immune responses during the effector and repair phases of respiratory virus infection. Here, we summarize reports of sex differences or sex hormone receptor regulation of innate immune cells. The pink shaded area indicates cells and pathways reported to be elevated in females and/or upon estrogen/ER activity. The blue shaded area indicates cells and pathways reported to be increased in males and/or upon androgen/AR activity. A balanced type 1 immune response involving different innate immune cells is required early post-infection in the lung for viral clearance. At later stages of infection, regulatory immune responses mediated by alveolar macrophages and innate lymphoid cells are important for the repair of damaged tissues and renewal of barrier integrity. Sex differences in numbers, functional responses, plasticity, and survival of innate immune cells regulate the proinflammatory/effector and regulatory/repair phases of infection.

Respiratory virus infections lead to significant health problems worldwide (1). Humans show marked sex differences in the severity, prevalence, and outcome of inflammatory lung diseases including viral infection (2, 3). Innate immune responses have crucial roles in early defense against viruses but also shape antigen-specific adaptive immune responses and promote tissue repair. A number of recent reviews highlight sex differences in innate immune pathways during infectious disease (4–6). Here, we review literature reports on the sex differences in numbers and functional responses of innate immune cells in the lung and their regulation by sex hormones in homeostasis and during viral lung infection. Specifically, we highlight ways in which sex differences in innate cells may influence both the proinflammatory/effector phase and the resolution/tissue repair phase important in the host response to respiratory virus infection (Figure 1).

## Sex Hormones and Receptor Signaling

### Sex Hormone Levels

Endogenous estrogens include estrone (E1), 17-β-estradiol (E2), and estriol (E3), with E2 being the major form present in adult females and males while E3 is present at high levels in pregnancy (7). Testosterone is synthesized in both females and males and is converted by 5α-reductase to the physiologically active metabolite dihydrotestosterone (DHT) or by aromatase to estradiol (8). Progesterone (P4) is present in both sexes and, in females, varies during the menstrual cycle and is produced by the placenta at high levels during pregnancy (9). The levels of circulating sex hormones vary in both sexes throughout the lifespan, with the highest levels of estrogens and progesterone in females and testosterone in males during the reproductive years (10, 11). In pregnancy, estrogens and progesterone reach significantly higher levels (8, 12). Sex hormone levels in humans in phases of the menstrual cycle and pregnancy are nicely summarized in a recent review (13). The dramatic changes in sex hormone levels at puberty are correlated with changes in immune function and susceptibility to immune-mediated disease. Sex hormones also are present *in utero* and immediately post-birth, and this may influence immune cell differentiation and neonatal immunity. The developing testes in male fetuses produce testosterone, and both sexes are exposed to high levels of maternal estrogens *in utero* (14, 15). In the first weeks after birth, both human and rodent males have a “mini-puberty,” in which testosterone levels approach those of adults (15–17).

Sex steroids are synthesized in the gonads and adrenal cortex, and in peripheral tissues such as liver, fat, and kidney (8, 18). Little information is available about local synthesis in the lung (8). Activated macrophages may increase local estrogen levels since cytokine receptor signaling induces their synthesis of aromatase, the enzyme that converts testosterone to estradiol (19). Few studies of immune cells in tissues have correlated tissue levels of sex hormones with immune function.

### Sex Hormone Receptors

Sex hormones mediate their effects through estrogen receptors (ERα and ERβ), androgen receptor (AR), and progesterone receptors (PR-A and PR-B) (20–22). Splice variants of ER leading to truncated but functional proteins such as ERα46 have been identified in myeloid cells (23). Sex steroid receptors are ligand-dependent transcription factors that recruit transcriptional coregulators such as SRC1 and histone-modifying enzymes such as p300/CBP into multi-protein complexes that bind DNA [reviewed in Ref. (20, 24)]. ERs, PRs, and AR bind to their respective response elements at specific DNA sites leading to epigenetic modifications of chromatin and changes in transcription of target genes. Nuclear sex hormone receptors also may be tethered indirectly to DNA *via* their ability to bind transcription factors such as SP1. Ligand-free receptors also can recruit corepressors such as NCOR and histone deacetylases to repress transcription. Rapid “nongenomic” sex steroid signaling occurs via inner plasma membrane-localized ER or AR, and possibly *via* the G protein-coupled receptor GPR30 (also termed GPER) (20, 25).

Innate immune cells express ERs (*Esr1, Esr2*), AR (*Ar*), and PRs (*Pgr*) to varying degrees. *Esr1* and *Ar* RNAs also are expressed at high levels in hematopoietic progenitors in bone marrow (BM), consistent with documented effects of sex hormones on immune cell differentiation and numbers in homeostasis (26). Based on our literature review and data from the Immunological Genome Project (www.immgen.org), Table 1 summarizes the relative expression of sex steroid receptor RNA or protein in hematopoietic progenitors and innate cells of the lymphoid and myeloid lineages. Since limited information is available about sex steroid receptor expression in lung-resident immune cells, Table 1 includes information for the cell type regardless of tissue location or activation state. Patterns of receptor expression may underlie the effects of the sex hormones on numbers and functional responses of innate immune cells. Some mature innate cells do not apparently express significant levels of the sex hormone receptors, but they may still function differently in the sexes due to epigenetic imprinting of developmental precursors or because their responses are influenced indirectly *via* other cell types responding to sex hormones.

**Table 1:** Expression of sex steroid receptors in human and murine innate immune cells.

Cell type	ERα	ERβ	Other ERs	PRs	AR	ERα	ERβ	PRs	AR
	
	Human						Murine			
Type II innate lymphoid cells						Yes (27)/No (28)	Yes (27)/No (28)	−/+	Yes (28–30)

Natural killer cells (NK)	Yes (31, 32)	Yes (31, 32)	ERα46 (31)	Yes (33)	−	Yes (34)	Yes (34)	−	−/+

Gamma delta T cells (γδ T)				Yes (35)		−/+ or +	−/+	−	+ or −

Natural killer T cells	−	−/+		−	−	Yes (36)	−/+	−	− or −/+

Neutrophils	Yes (37)	Yes (37)	GPER (38)	No (39)	Yes (40)	−/+	−	−	Yes (41)

Eosinophils	−/+	−/+	GPER (42)	No (39)	No (40)	−/+	−	−	−

Plasmacytoid dendritic cells (pDC)	Yes (32)	Yes (32)		−	−	Yes (43)	−	−/+	−

Monocytes	Yes (23, 32, 44, 45)	Yes (23, 32, 44, 45)	ERα46 (23)	−	−	+	−/+	−	−

Dendritic cell (tissue-resident, monocyte-derived, BM-derived)	Yes (32, 45)	Yes (32, 45)		−	−	Yes (46, 47)	Yes (46, 47)/No (48)	−/+	−

Macrophage (alveolar, BM-derived, peritoneal)	Yes (23, 49, 50)	Yes (23, 49, 51)	ERα46 (23)	Yes (49)	Yes (40, 52)	Yes (48, 53)	Yes (50)/No (48, 53)	Yes (54)	Yes (55)

Hematopoietic stem cell	Yes (26)	Yes (26)		−	Yes (26)	Yes (56)	No (56)	No (56)	No (56)

*The presence of sex steroid receptors in each cell type (located in any tissue and regardless of activation state) is indicated by “Yes” or “No” and the literature reference. Some cell types differ in receptor expression in different tissues, and this is indicated by “Yes/No.” If a literature report was not found, we consulted the Immunological Genome Project, and the presence or absence of receptor RNA is indicated in blue if available*.

*−, <50 counts; −/+, 50–100 counts; +, 100–300 counts; + or − represents positive or negative value depending upon the tissue location*.

Sex differences in lung development, structure, and function have been identified (57). The lungs of human females are smaller than males of the same height; however, airway size and capacity do not always correlate with lung size, and the extent and type of sex differences in lung function vary throughout the lifespan (58). Smooth muscle, fibroblasts, and epithelial cells express sex steroid receptors (8), and their functional responses in different sex hormone environments may alter the immune response or modulate infection severity.

### Methods to Study Effects of Sex Hormones and Receptors on Immunity *In Vivo*

Investigators have taken multiple approaches to understand the impact of sex differences and sex hormone receptor signaling on immunity. Diverse approaches in different experimental models have often led to conflicting results. Age should be carefully considered when studying sex differences in immune cell numbers or functional responses in humans and rodents since sex hormone levels vary over the lifespan (2). Littermate or colony matching will reduce variables such as diet and microbiome and help to identify sex differences. Studies of sex differences in human immunity would be improved by direct measurements of hormone levels in each individual (particularly in women) because age alone does not predict hormone levels modulated by oral contraceptives or hormone replacement therapy. A common approach is to gonadectomize young mice and replace estrogens or androgens by implantation of time release pellets. While this strategy has led to many answers, the absence of sex hormones in young gonadectomized mice may alter immune cell development and numbers prior to infection. In addition, replacement of hormones to a constant level does not mimic the cyclic variation that occurs particularly in females. Similarly, it is difficult to recapitulate accurate *in vivo* exposures of sex hormones in cell culture models. Another approach is to impose male levels of DHT in a female mouse (or female levels of estradiol in a male mouse) to help elucidate sex hormone interactions and their effects independent of chromosomal sex and developmental programming.

Mice lacking sex hormone receptors also have informed our understanding of sex differences in immunity. However, global deletion of sex hormone receptors can lead to abnormal levels of estrogens and androgens; for example, global *Esr1* deficiency leads to high levels of circulating testosterone (59–61). Furthermore, global loss of receptor signaling may alter the function of non-immune cell types in ways that impact immune responses. To circumvent this issue, investigators are beginning to study mice bearing conditional deletion of *Esr1* or *Ar* along with lineage-restricted Cre drivers to understand the effects of sex hormone receptor deficiency on numbers and function of specific cell types. This approach will help to identify direct effects of sex hormone receptor signaling in immune cells. Use of emerging technologies such as single cell RNA-sequencing, assay of transposase-accessible chromatin-sequencing, multiplex mass cytometry, or chip cytometry (62–64) to monitor the transcriptome, epigenome, or proteome at the single cell level will help us to understand sex differences in immune function and how sex hormone receptors regulate immune cells in homeostasis and during viral infection. These approaches will be especially valuable to dissect the diversity of responses of rare immune cell types in peripheral tissues such as the lung. More precise methods and attention to age and hormonal cycles and levels will help to clarify the roles of sex hormones and receptors in immune responses.

## Sex Differences in Innate Immune Responses in Respiratory Virus Infection

Despite the lung’s structural and chemical barriers to pathogen entry, many viruses subvert these barriers and efficiently infect and replicate within lung epithelial cells [reviewed in Ref. (65)]. Damage to host lungs may be directly induced by the virus or be secondary to a strong immune response. Upon respiratory virus infection, immune cells typically participate in three phases: (i) innate immune cells sense presence of the virus and initiate early antiviral responses and prime the adaptive response; (ii) effector or adaptive immune cells clear virus by killing infected cells and producing antiviral antibodies, followed by conversion of a subset to memory lymphocytes; and (iii) innate immune cells act in concert with epithelial regeneration pathways to repair injured tissue and produce mediators that return the immune system to homeostasis (65). Herein, we focus on responses of innate immune cells in the initiation and repair phases of respiratory virus infection.

Epidemiological studies of humans and experimental models with rodents show that it is difficult to arrive at a universal paradigm regarding effects of sex or sex hormones on immune responses to respiratory viruses. vom Steeg and Klein hypothesize that sex differences in infection outcome are a function of the strength of the immune response and resulting host tissue damage (66). In this model, a male bias in risk occurs when weak immune responses underlie significant host damage, while a female bias in risk occurs when strong immune responses promote host damage. Experimental manipulation of sex hormones and their receptors in rodents has shown that sex differences in systemic estrogen and androgen levels often underlie differential immune function and infection outcome. Depending on the role of the sex hormone to promote or inhibit inflammation or immunity, sex differences may arise due to either the predominance of, or the absence of, estrogen or androgen in one sex.

In the initiation phase, lung-resident dendritic cells (DCs) and macrophages (alveolar and interstitial) respond to viral molecules (nucleic acids and glycoproteins) *via* cell surface or intracellular receptors that are linked to signaling pathways resulting in production of interferons (IFN), cytokines, and chemokines (65, 67). Coupled with these viral “pathogen-associated molecular patterns,” damage to host cells results in the release of host molecules such as ATP, heat shock proteins, or HMGB1, termed “danger-associated molecular patterns,” which also trigger innate immune receptors and inflammasomes. Innate lymphocytes respond to cytokines produced by activated myeloid cells or alarmins released by damaged tissue and in turn produce type 1 (IFNs, IL-12, IL-1β, TNFα) or type 2 or regulatory (IL-5, IL-10) cytokines that direct subsequent innate or adaptive responses. Type I and III IFNs elicit expression of molecules that are directly antiviral. DCs acquire and present viral antigens, migrate to draining lymph nodes and prime adaptive responses through interactions with naïve T. Activated T cells then return to the lung where they interact again with resident or recruited myeloid cells, produce pro- or anti-inflammatory cytokines, and lyse infected cells.

Respiratory viruses typically elicit strong type 1 immune responses involving myeloid cell production of type I and III IFN and proinflammatory mediators such as IL-12, TNFα, and CCL2 and lymphocyte production of IFNγ (67). As described in detail in later sections, there is some evidence for sex differences in (or sex hormone regulation of) the function of myeloid cells and innate lymphocytes during respiratory virus infection. However, more often, reports of sex differences or sex hormone regulation involve studies of immune cells at other tissue sites, in autoimmune or other pathogen models or performed *in vitro*. In brief, type I IFN synthesis is promoted by estrogen and ERα signaling, and multiple reports show that female plasmacytoid DCs (pDCs) produce more type I IFN than male pDCs (32, 43, 68, 69). Sex hormone regulation of proinflammatory cytokines (IL-12, IL-6, IL-1β) seems more complex, but a number of studies show that lower physiological levels of estrogens enhance their production while higher physiological levels dampen their production and instead promote regulatory cytokines such as IL-10 [reviewed in Ref. (24)]. In contrast, reports show that testosterone decreases cytokines such as IFNγ and TNFα and increases IL-10 (21).

Regulatory and type 2 immune responses are important in later stages of respiratory viral infection. It is now recognized that while type 1 responses are important for viral clearance, type 2 responses also are elicited and promote repair of injured tissue and resolution of the immune response upon influenza virus and respiratory syncytial virus (RSV) infection (70–72). In murine models of allergic asthma, estrogen and ERα signaling promote type 2 responses of alveolar macrophages (AM) (73), while androgens and AR signaling attenuate type 2 responses promoted by innate lymphoid cells (ILC2s) and myeloid cells (74). These ER and AR regulated pathways also may be important in respiratory virus infection. Indeed, the chronic elevation of type 2 responses in asthmatic individuals can lead to a milder course of influenza virus infection and reduced lung injury (75), while ILC2 activity in influenza virus infection can exacerbate asthma (76).

### Influenza Virus A (IAV) Infection

Sex differences in the incidence and severity of IAV infection in the human population have been well documented (13, 77). However, given the strong impact of age on morbidity and mortality, it is often difficult to separate effects of sex and age since sex hormone levels change dramatically with age (78). Furthermore, social and cultural differences in gender norms also may influence ascertainment or self-reporting of infection symptoms or access to medical care. While the incidence of infection is often higher in males, females often show greater morbidity. Increased infection severity in females may result from stronger innate and adaptive responses in females that lead to more extensive immunopathology. Epidemiological studies from the 1957 H2N2, 2005 H1N1, and 2009 H1N1 pandemic IAV infections showed that the mortality and hospitalization of patients following viral infection was higher for females than males during their reproductive years (77, 79–81). This suggests that adult levels of sex hormones modulate immunity to IAV infection; however, these studies did not measure immune responses at the molecular or cellular level. Females in their reproductive years also have increased asthma incidence (58), which may alter immune responses and increase IAV-induced pathology. In contrast, infection of young males (<age 20) and elderly adults (>age 80) led to greater hospitalization or mortality (80). While this might suggest that lower levels of androgens in young boys and elderly men correlate with increased infection severity, information about comorbidities and measurement of androgen levels coupled with more precise information regarding susceptibility of males pre- and post-puberty would be needed to make this correlation.

We also lack information regarding differential susceptibility to IAV infection in distinct phases of the menstrual cycle and in women taking oral contraceptives. These hormonal variables may modulate susceptibility or severity of IAV infection, as epidemiological data from asthmatic women and girls show premenstrual aggravation of asthma symptoms and alleviation of this cyclical effect while taking the oral combined contraceptive pill (58). Pregnancy was highly associated with increased mortality and morbidity following IAV infection, and one factor may be immune suppression by elevated estrogens and progesterone (82–84).

Studies of mice infected with mouse adapted and pandemic H1N1, and avian H3N1 and H7N9, viruses have provided valuable insights into sex differences in susceptibility and immunity to IAV. Morbidity, mortality, and the associated inflammatory response is greater in female than male mice at moderate IAV loads, but mortality of both sexes is similar at higher loads (85–87). At sublethal doses, females showed higher levels of TNFα, IFNγ, and CCL2 (85, 88) and neutralizing anti-influenza antibodies, which correlated with greater protection upon heterosubtypic virus challenge (86). At viral doses lethal in females, but not males, estrogen protected from mortality, as shown by comparing ovariectomized mice supplemented with estradiol or placebo (85). Estrogen replacement correlated with reduced TNFα and CCL2 production, yet increased numbers of neutrophils and CD8^+^ viral antigen-specific T cells producing IFNγ (89). Overall, gonadally intact and gonadectomized females produced greater inflammatory responses and showed increased morbidity following infection, suggesting that low levels of estrogens promote excessive inflammatory responses. In contrast, replacement of higher levels of estradiol to gonadectomized mice ameliorated inflammation and promoted adaptive immunity. This is consistent with anti-inflammatory effects of replaced estrogen in autoimmune disease models (90) and the ability of ERα to negatively regulate NF-κB signaling [reviewed in Ref. (20)].

Ovariectomy of females followed by progesterone replacement to luteal phase levels also reduced morbidity upon IAV infection (91). Progesterone led to increased tissue repair due to upregulation of the epidermal growth factor amphiregulin (Areg) in the lung (91). These studies suggest that progesterone-based contraceptives may promote recovery from respiratory virus infection (9).

Gonadectomy of young males increased morbidity and pathology upon IAV infection, and replacement of testosterone or DHT, which cannot be metabolized to estradiol, reduced morbidity, mortality, and inflammation (85, 92). In contrast, testosterone treatment of old male mice, which have decreased testosterone levels as in humans, increased survival but did not alter pathology (92). These data are consistent with the ability of testosterone to suppress inflammation (21, 93).

In murine models of sublethal IAV infection, morbidity typically is most related to immune-mediated pathology rather than failure to clear virus (67). Thus, data from the above studies suggest that the increased morbidity and mortality of females is secondary to a strong proinflammatory response that leads to extensive tissue damage, while the lesser morbidity in males is the result of a more balanced immune response that clears virus with less tissue damage. Sex hormones that suppress inflammation (testosterone, progesterone, or high levels of estrogens) may attenuate antiviral immune responses to an optimal level, while lower levels of estrogens and androgens may permit excessive inflammation in some cases. The evolutionary benefit of this disparate effect of female and male sex hormones on immunity remains unclear.

### Other Respiratory Viruses

Infection by two other respiratory viruses leads to increased morbidity in males. RSV is a common respiratory tract infection that most often progresses to the lower respiratory tract with severe consequences in infants and the elderly. The overall incidence of RSV is higher in young boys than girls (94, 95); however, the possible immunological basis of this sex difference and the role of sex hormones remains unknown. In outbreaks of pathogenic coronaviruses including the severe acute respiratory syndrome (SARS-CoV) and the Middle East respiratory syndrome (MERS-CoV), males showed increased infection incidence and severity (96, 97). Male mice showed enhanced susceptibility to SARS-CoV including elevated viral titers and increased accumulation of inflammatory monocytes and neutrophils in the lungs (98). ER signaling in females may be protective in this infection since ovariectomy or treatment with an ER antagonist increased mortality, while male gonadectomy did not alter disease outcome.

## Sex Differences in Innate Immune Cells During Respiratory Virus Infection

During respiratory viral infection, responses of lymphoid and myeloid innate cells play a crucial role in early antiviral protection and promote the generation of adaptive immune responses including effector and memory T and B cells. Here, we highlight studies demonstrating sex differences and effects of sex hormones in the number, function, and development of innate cells in the respiratory tract (Figure 1). We also review reports of sex differences and sex hormone regulation in innate cells in other tissues, which may inform our understanding of sex-dependent regulatory mechanisms in the respiratory tract. This topic is the subject of excellent recent reviews (74, 93, 99, 100).

## Innate Lymphocytes

### Type II ILC2s

Innate lymphoid cells are tissue-resident cells that develop from lymphoid progenitors but lack antigen specific receptors. Like T cells, ILCs are divided into the ILC1, ILC2, ILC3, and natural killer cell (NK) subsets based on expression of fate-determining transcription factors and cytokine production (101). In homeostasis, ILC2s are the predominant ILC subset in the murine lung (102), and both ILC2s and ILC3s are predominant in human lung (103). NKs (see below), ILC1s, and ILC2s generate innate responses during IAV infection while the role of ILC3s has not been investigated. Notably, ILC2s in murine lung, BM, and small intestine express high levels of *Ar* but little *Esr1* or *Esr2* (27–30).

Respiratory viral infections cause death of lung epithelium mediated by viral toxicity and immune cell activation, and appropriate remodeling of lung tissue to maintain barrier integrity is crucial (70). ILC2s are important for tissue repair following IAV infection as they expand and secrete Areg, IL-13, and IL-5 (76, 104, 105). Areg promotes regeneration of the bronchial epithelium, and IL-13 promotes barrier integrity by inducing epithelial cell proliferation and survival (91, 106). IL-5 recruits eosinophils that promote antiviral immunity and lung tissue regeneration in the resolution phase (105, 107, 108). *Via* these pathways, ILC2s facilitate tissue repair in IAV and RSV infection (104, 109).

We and others reported sex differences in murine lung ILC2 numbers, with female mice harboring more lung ILC2s compared to males in homeostasis (28, 30, 110). A functional subset of lung ILC2s that lack the inhibitory E-cadherin-binding receptor KLRG1 is uniquely present in females (28, 110). Experiments involving hormone replacement in gonadectomized mice and mice bearing global or conditional deficiency in *Esr1* or *Ar* showed that the sex difference in ILC2s is regulated by androgens and AR but not estrogens or progesterone (28, 30, 110). Males have increased numbers of ILC precursors in BM, suggesting that androgens attenuate the progression from ILC precursor to mature ILC2 (110). In humans, sex differences in lung ILC2s have not yet been investigated; however, increased numbers of ILC2s are present in the blood of asthmatic females compared to males (30). Interestingly, sex hormones may regulate ILC2s differently in each tissue. Estrogen and ERα signaling sustain uterine ILC2s, which express high levels of *Esr1* compared to lung ILC2s (27). Fewer ILC2s accumulate in the central nervous system of female mice in the EAE model of multiple sclerosis (111). A lower proportion of ILC2s are present in cord blood of human female neonates compared to males (112).

Innate lymphoid cells in gonadectomized males produce more IL-5 and IL-13 after stimulation (28). Similarly, DHT treatment *in vivo* decreases IL-5 and IL-13 production by ILC2s (30), although a direct role for AR was not tested in these studies. Together with the finding that progesterone increases Areg expression (91), these data suggest that IAV-infected females may show superior tissue repair due to increased numbers of ILC2s capable of producing IL-13, IL-5, and Areg.

Alternately, the higher number of ILC2s in females may induce more pathology due to their functional plasticity. ILC2s convert to ILC1-like cells producing IFNγ in response to IL-12 and IL-18 produced during IAV infection and lung inflammation triggered by smoking or chronic obstructive pulmonary disease (113–115). Although sex differences in ILC2 plasticity during IAV infection have not been reported, higher numbers of ILC2s that are capable of converting to ILC1s at the peak of infection may contribute to more severe immunopathology in females.

### Natural Killer (NK) Cells

Natural killers are cytotoxic innate lymphocytes that control viral burden *via* their early production of IFNγ (116). NKs enhance DC migration and T cell recruitment upon infection with a sublethal IAV dose, but depletion of NKs was protective after infection with a lethal dose (116). Human and murine NKs express ERs and PR but not AR (Table 1). Human studies revealed higher numbers and cytotoxic activity of blood NK cells in males compared to females (117–119). This sex difference was reversed in old age (120). Studies show that NK numbers in blood correlate with stages of the menstrual cycle, suggesting regulation by sex hormones (121–123). In pregnancy, the recruitment of NKs from the blood to the uterine mucosa coincides with the dramatic rise in estriol and progesterone (124, 125). However, the effect of estrogen or progesterone on NK cell activity is unclear since some studies showed that *in vitro* (human) or *in vivo* exposure to estrogen or progesterone decreases NK cell activity while others found no effect (33, 126–129). Sex differences in NK numbers or function during IAV infection have not been reported.

### Gamma Delta (γδ) T Cells

Innate γδ T cells bear TCRs with limited junctional diversity that recognize intact protein antigens and small phosphate or amine containing molecules (130). γδ T cells are divided into different tissue-specific subsets based on predominant pairings of particular Vγ or Vδ genes (131). γδ T cells show important functional responses during infection by RSV and IAV. In murine RSV infection, γδ T cells are recruited to the lungs and produce IFNγ, IL-17A, IL-10, and IL-4 resulting in the activation of other innate cells (132). Vγ4^+^ T cells also secrete IL-17A during IAV H1N1 infection to aggravate acute lung immunopathology (133). Levels of circulating Vγ9/Vδ2 T cells in adult women were significantly higher than in men (134); however, another study showed the opposite trend (135). Sex differences in γδ T cells in mice have not been reported.

### Natural Killer T Cells (NKT)

Natural killer T cells facilitate cross-talk between the innate and adaptive immune system during viral infection. NKT cells are a subset of T lymphocytes expressing a restricted αβ TCR that recognizes CD1d-bound lipids. NKT cells play a protective role in IAV infection through their secretion of IL-22 and IFNγ to activate NK cells and CD8^+^ T cells (67). The absence of NKTs in a murine model of RSV infection led to a delay in viral clearance, suggesting a protective role in infection (136). In mice, estrogen acting *via* ERα regulates a sexual dimorphism in NKT function. Administration of estradiol to ovariectomized mice increased NKT IFNγ production upon *in vivo* stimulation by IL-12 + IL-18 and a CD1d ligand, and NKTs in ERα−/− mice produced less IFNγ (36). Reports of sex differences in human NKTs are inconsistent, and data from the Immunological Genome Project show only low levels of sex hormone receptor RNA in human NKTs (Table 1). Increased blood NKT cell numbers in women relative to men was reported in some studies (137–139). Sex differences in NKT cells in respiratory virus infection have not been reported, but in view of the above studies, analyses of possible sex disparate responses in NKT function in murine models of IAV or RSV may yield important insights.

### Innate Lymphocyte Summary

Innate lymphocytes express sex hormone receptor RNAs at varying levels depending on the subset and tissue location (Table 1). While NK and NKT cells primarily express ERs, lung ILC2s predominantly express AR, suggesting regulation of the classes of innate lymphocytes by distinct sex hormone-mediated mechanisms. However, we lack information about sex differences in numbers and function of these diverse subsets in the murine lung during respiratory virus infection. Recent studies have shown a profound sex difference in numbers and functional responses of murine lung-resident ILC2s, and AR signaling decreases numbers of ILC2s in males. Future work will determine if this numerical disparity in ILC2s leads to sex differences in the resolution of respiratory virus infection. Reports of sex differences in numbers or function of innate lymphocytes in human blood are often conflicting, and more studies that carefully correlate gender, age, and sex hormone status with lymphocyte numbers and function in blood or tissues are needed to clarify the field.

## Myeloid Cells

### Neutrophils

Neutrophils are the predominant infiltrating innate cell type during respiratory viral infection in both humans and mice. Neutrophils mediate antiviral defense *via* their production of proinflammatory cytokines and reactive oxygen species (140). Their role in respiratory viral infection remains unclear, as they cause pathology and susceptibility to secondary infections in mice. Neutrophil numbers and neutrophil extracellular trap (NET) formation directly correlate with the severity of RSV infection (141, 142).

Neutrophils express ER and AR (Table 1), and sex differences in the number and function of neutrophils in humans have been reported. Neutrophil numbers in blood are increased during pregnancy and the luteal phase of the menstrual cycle, suggesting that higher levels of progesterone or estrogens promote neutrophil numbers (143–145). Neutrophils from healthy women of reproductive age show improved survival *in vitro* compared to those of healthy men. Estradiol and progesterone contribute to the delay in neutrophil apoptosis by decreasing expression of the pro-apoptotic protein caspase 3 (146). Other studies showed that sex hormones modulate neutrophil function *in vitro*, including chemotaxis and nitric oxide and superoxide production (147–149).

Sex hormones also regulate neutrophil numbers in homeostasis and infection in murine models. AR-deficient mice show reduced numbers of neutrophils and neutrophil precursors in BM (41). Consistent with regulation of neutrophil numbers by AR signaling, the enhanced susceptibility of male mice to SARS-CoV infection was associated with increased accumulation of neutrophils in the lung (98). In contrast, estradiol treatment of ovariectomized females elevated neutrophil chemoattractants and recruitment of neutrophils into the lungs, thereby increasing protection in IAV infection (89).

### Eosinophils

Eosinophils enhance antiviral immunity during RSV infection by sensing viral RNA *via* TLR7 and producing nitric oxide (150). In IAV infection, eosinophil degranulation and activation of viral antigen specific CD8^+^ T cells increases protection against infection (151). Estrogen increases eosinophil migration adhesion, survival, and degranulation both *in vitro* (42, 152) and *in vivo* (153). Furthermore, the number of eosinophils in female rats peaks with higher levels of estrogen during estrus, and ovariectomy significantly reduces uterine eosinophils (154, 155). These studies suggest that female sex hormones regulate eosinophil numbers, but sex differences in the numbers or function of eosinophil during respiratory viral infection have not been reported. Since eosinophils were reported to express very little *Esr1* and no *Ar* or *Pgr* RNA (Table 1), sex differences in eosinophil numbers may be secondary to the sex differences in numbers of IL-5-producing ILC2s.

### Alveolar Macrophages

Alveolar macrophages are lung-resident phagocytic cells that induce protective antiviral immune responses *via* production of soluble mediators (156). In viral infection, AMs produce high levels of type I IFN important for viral clearance and chemokines that recruit inflammatory monocyte into the lung (156). Sex differences or the effect of sex hormones in AM function during respiratory virus infection have not been described, although murine AMs express both ERα and AR (73). Studies of peritoneal macrophages, which also express ERβ, offer some insight into how AMs may be regulated by sex hormones during virus-induced inflammation. Increased numbers of macrophages were present in the pleural and peritoneal cavities of female mice, and they showed higher levels of TLRs and phagocytic capacity, which was associated with stronger acute inflammatory responses (157). Consistent with this, inflammatory TLR-mediated responses of human monocyte-derived macrophages and murine peritoneal macrophages were enhanced by estrogen and reduced by testosterone exposure (158–161).

The roles of sex hormones in AM function during allergic asthma may provide insight into sex differences in AM functional responses in viral infection. AMs are polarized to an M1 phenotype in a type 1 environment involving IFN or to an M2 phenotype in a type 2 environment involving IL-4/IL-13. In allergy models, female mice show an increased type 2 polarized AM response, and estrogen signaling *via* ERα in AMs was an important driver of the allergic response *in vivo* (73, 99, 162). This is consistent with other reports that female sex and/or ERα signaling promotes M2 macrophage function in cutaneous wound healing (163), Coxsackievirus-induced myocarditis (164), and atherosclerosis (53). In contrast, AR activity in macrophages suppresses wound healing by enhancing local TNFα expression (165). These data suggest that estrogens and ERα may promote, while AR may attenuate, the type 2 responses that promote tissue repair in the resolution phase of a viral infection.

### Monocytes and Monocyte-Derived Cells

Monocytes respond to viral infection by secreting cytokines and chemokines. They also are precursors to “inflammatory” macrophages or DCs in tissues. Following virus infection, CCR2^+^ monocytes are recruited *via* the chemokine CCL2 from blood to the lung, where they differentiate into DC- or macrophage-like cells often producing proinflammatory cytokines such as TNFα and IL-12 (166). Physiological levels of estradiol decrease expression of CCR2 and CXCR3 on murine monocytes *in vivo*, suggesting that ER signaling might reduce monocyte recruitment to tissues (167). Indeed, systemic estradiol treatment of ovariectomized mice reduced CCL2 induction and numbers of infiltrating monocytes during IAV infection, although no differences in numbers of inflammatory monocyte-derived DCs (Mo-DCs) were noted (89). Consistent with this, SARS-CoV infection of more susceptible male mice led to increased accumulation of monocyte-derived cells (Ly6C^+^ CD11b^+^) producing proinflammatory cytokines relative to female mice, and depletion of the monocyte-derived cells partially protected mice from a lethal infection (98). In this model, ovarian hormones and ER signaling in female mice were protective while orchidectomy of male mice had no effect, suggesting estrogens rather than androgens regulate pathogenic monocyte responses.

Reports of sex differences in human monocyte numbers and cytokine production are inconsistent and may reflect the diversity of the human population. Postmenopausal women showed increased numbers of monocytes compared to premenopausal women (168). Other work showed that monocyte counts were higher in the luteal phase associated with higher progesterone levels than in the follicular phase (143). Pregnancy also was associated with higher monocyte numbers, yet reduced capacity for IL-12 and TNFα production (169). Peripheral monocytes from healthy females produced more IL-6 upon LPS stimulation as compared to males (170). However, studies to determine if estrogens regulate pro-inflammatory cytokine production by female monocytes and monocyte-derived macrophages showed either negative (167, 171) or positive regulation (172). Macrophages and monocytes exposed to testosterone decreased their production of proinflammatory cytokines and increased synthesis of IL-10 (173–175).

### Dendritic Cells

Dendritic cells are professional antigen-presenting cells classified by phenotype and functional capacity into distinct subsets including (pDCs), conventional DCs (cDCs), and Mo-DCs. While the lung harbors at least three subsets of tissue-resident cDCs (176), pDCs and Mo-DCs enter the lung in significant numbers upon infection. Murine lung-resident DCs express *Esr1* but little *Ar* (Table 1). The direct effect of sex hormone receptor signaling in these DC subsets in the lung during respiratory virus infection has not been reported. However, studies of sex differences and sex hormone effects on DCs in other tissues may provide some clues about lung DC subsets (100, 177).

Upon infection, lung-resident cDCs migrate to the draining mediastinal lymph nodes and prime naïve T cells. While sex differences in the numbers or function of these DCs during virus infection have not been reported, no differences in lung cDC numbers were found in ovariectomized mice treated with placebo or estradiol and infected with IAV (89). Functional studies with murine BM-derived DCs showed that estradiol and ERα signaling promote the TLR dependent production of proinflammatory cytokines of cDCs in the Flt3L-driven model and inflammatory DCs in the GM-CSF model (178–181). Estradiol also increased the production of IL-8 and CCL2 from human Mo-DCs (182). Other studies have shown that estradiol promotes GM-CSF-driven DC differentiation *in vitro* [reviewed in Ref. (177)]. Estradiol acts *via* ERα in murine myeloid progenitors to promote DC differentiation by upregulating the transcription factor IRF4 (183). In contrast, progesterone decreased TNFα and IL-1β but not IL-10 production by rat BM-derived DCs (184) and reversed estradiol-mediated changes in differentiation and function of BM-derived murine DCs (185). Progesterone modulated TLR-induced activation and cytokine production by murine BM-derived DCs (186).

Plasmacytoid DCs rapidly respond to viral particles *via* endosomal and cytosolic sensors of viral nucleic acids and produce type I IFN and IFN-induced proteins that are directly antiviral. Female pDCs produce significantly more IFNα in response to viral nucleic acids or synthetic TLR7 ligands than male pDCs (68, 69), and this correlates with higher levels of ERα-regulated IRF5 in female cells (187). Estrogen signaling and XX chromosome dosage promoted sex differences in TLR7-mediated IFNα production by human pDC (32), and estradiol treatment of postmenopausal women enhanced their production of IFNα (43). Models of conditional *Esr1* deficiency in DCs showed that ERα signaling drives sex differences in pDC functions (43, 188). Consistent with greater production of type I IFN by pDCs or other innate cells, female rats infected with respiratory Hantavirus showed greater expression of genes encoding viral nucleic acid sensors and type I IFN compared to males (189).

Testosterone and progesterone may suppress pDC responses, although pDCs do not apparently express significant levels of *Ar* or *Pgr* RNA in homeostasis (Table 1). Progesterone inhibits IFNα production by pDCs (190). Upon stimulation with a TLR7/8 agonist, human infant male infant pDC responses were significantly lower than those of females (191), which may be due to increased testosterone (or lower estrogen) levels in infants post-birth. Male PBMCs produced similar amounts of IFNα, yet greater amounts of IL-10 than female PBMCs upon IAV stimulation, and the IL-10 may dampen type 1 inflammation in males (192, 193). Taken together, these studies show that female pDCs produce higher levels of type I IFNs, consistent with stronger antiviral immune responses, yet more immunopathology in females.

### Myeloid Cell Summary

Sex differences in the numbers or functional responses of myeloid cells in murine models of IAV and coronavirus infection have been reported. Manipulation of sex hormone signaling through gonadectomy −/+ sex hormone replacement, or ER or AR deficiency, has provided evidence for sex hormone-mediated regulation of neutrophils, pDCs, monocytes, and monocyte-derived cells in the lung during infection. Sex differences in lung-resident cDCs during infection have not been reported, but these DCs do express *Esr1* suggesting estrogens may regulate their important role in initiation of innate and adaptive responses to viruses. In asthma models in which females exhibit more disease, sex hormones regulate AM type 2 responses, suggesting that sex differences in AM function during the resolution phase of respiratory virus infection also may occur. Overall, more research is needed to fully understand mechanisms of sex hormone regulation of myeloid cells during respiratory virus infection and how these may contribute to sex differences in antiviral defense.

## Concluding Remarks

Sex differences in immunity to respiratory viruses are evident in humans and experimental rodent models. Sex hormones may act directly in innate immune cells or their precursors to promote or attenuate their function, but it is also probable that innate cells are indirectly modulated by actions of other immune or non-immune cells responding to sex hormones. Differential regulation of innate cells by sex hormones during the proinflammatory/effector phase and resolution/repair phase is likely to shape the mechanisms of viral clearance and the host capacity to resolve inflammation and repair damaged tissue. For example, estrogens and ER signaling may promote IFN production by pDCs and NKT cells early post-infection, but also type 2 or regulatory responses of AMs important for optimal resolution of the infection. Sex or sex hormones may not have universal effects during respiratory virus infection. Indeed, although endogenous estrogens in gonad-intact murine females promoted inflammation during IAV, they were protective in coronavirus infection.

While elegant studies of sex differences and the role of sex hormones have informed the field of innate antiviral immunity, we still lack information on how sex hormone receptors act in individual cell types to regulate functional responses. Many reports of sex differences or sex hormone effects in immunity are conflicting, most likely because of experimental approaches that do not fully take into account sex hormone levels varying due to age or cycle, difficulty in reproducing natural sex hormone levels *via* manipulation *in vitro* or *in vivo*, or hormone imbalances in globally *Ar* or *Esr1* deficient mice. Our understanding of sex biases in the antiviral responses of innate lymphoid and myeloid cells of the respiratory tract will be greatly facilitated by more precise approaches and measurements enabled by emerging technologies. When possible, careful studies of innate immune cells in the respiratory tract of infected humans would also contribute greatly to our understanding of sex-specific molecular and cellular pathways that underlie population data on incidence and severity of viral infections.

Whether sex differences in immunity confer an advantage at the population level remains unclear. Ideally, the capacity for strong immune responses to infection or tumors would be balanced by a lesser propensity for autoimmunity. Studies suggest this continuum differs between the sexes, with females often capable of superior immunity to pathogens but more susceptible to autoimmunity (2), although not all reported data fit into this simple model. Sex differences in immune function may arise as a byproduct of the distinct levels of androgens and estrogens that specify biological sex and gonad development. Consistent with their ability to bind DNA and regulate chromatin conformation, sex hormone receptors may act early in the pre- or postnatal period or during puberty to imprint sex-specific epigenetic patterns in the genome (5, 20). Epigenetically imprinted regions of open or closed chromatin in hematopoietic progenitors may differ between the sexes, and a sex divergent epigenome may be reinforced in mature immune cells in response to the sex hormone environment. The challenge of the field is to understand how sex hormones and their receptors regulate the epigenome and transcriptome in innate immune cells to mediate sex-divergent pathways that govern antiviral immune responses.

## Author Contributions

S Kadel and S Kovats reviewed the literature and wrote the manuscript.

## Conflict of Interest Statement

The authors declare that the research was conducted in the absence of any commercial or financial relationships that could be construed as a potential conflict of interest.
